# Translations

**Published:** 1881-12

**Authors:** Seth N. Jordan

**Affiliations:** Columbus, Ga.


### Translations.

By SETH N. JORDAN, M.D., Columbus, Ga.

*From Foreign Journals for the Atlanta Medical Register.*

*Naphthol Again. Kaposi. Med. Press*, No. 22–24, 1881.—Naphthol is chemically pure tar; it is soluble in its own weight of alcohol, in oil, and in fats. Applied to the normal skin it produces only an agreeable pliancy. Concentrated solutions, when applied repeatedly, cause the skin to turn brown and to peel off. Irritation can also follow its continuous use in strong solutions. An ointment 1 to 15: 100 can be used steadily for from 4 to 7 days.

Naphthol is resorbed by the skin and expelled by the kidneys. After employing it 12 hours the urine gets cloudy, sometimes of an olive green, resembling carbolic urine. In one single instance of a child with prurigo, after applying a 5 per cent. naphthol ointment for two days, a violent inflammation of the kidneys was set up. Child recovered; after which the same ointment was used on him again for four weeks without any unfavorable symptoms developing. Naphthol in solution and in ointments is scarcely perceptible to the smell. Linen is colored slightly pink by it, but can be washed out entirely. Kaposi has treated 106 patients with it. 52 patients were affected with scabies. Each patient was anointed twice in 24 hours with the following ointment: ℞—Naphtholi 15.0=ʒiv, axung 100.0=℥iii, sapon. virid. 50.0=℥iss, cret. alb. 10.0 ʒiiss.. This ointment has all the good qualities of the Hebra

Wilkinson ointment, a very popular ointment in the Vienna clinique. In every case a complete cure of the pustules and other effects of the parasite was made. Seventeen cases of psoriasis were treated by Kaposi with naphthol. The results were fully equal to those obtained from chrysophanic and pyrogallic acids, and with the difference of non-irritation of the skin and non-discoloration of both skin and linen in favor of naphthol. In eczema naphthol can be applied only under the same conditions that we employ tar. If the time in the stage of the eczema is chosen in which no longer a lively inflammation of the skin exists, then 2–4 inunctions of a $\frac{1}{2}$–2 per cent. solution of naphthol will render the skin pale, smooth and pliant. Should, however, desquamation take place, or even discoloration of the skin, the naphthol should at once be discontinued. Of especial value is naphthol in artificial eczema, as in consequence of prurigo and scabies. Kaposi emphasizes the beneficial influence of naphthol in cases of seborrhœa capilitii, prurigo, eczema squamosum et seborrhoicum, icthyosis, lupus erythematodes. In lupus vulgaris and epithelioma naphthol is inert. In conclusion, Kaposi adds that in naphthol we get all the advantages of tar stripped of its disadvantages.

---

*Resorcin.*—Part II. *By Dr. Justus Andeer, Centralblatt f. Med. Wissenschaften.*—I. Diseases of the Uterus.—Although the bladder of all the cavernous organs of the body tolerates resorcin the best when injected in large quantities, the opposite of this takes place in the case of the uterus. A two per cent. solution of this drug, either warm or cold, injected into the uterus, produces marked reflex phenomena. On this account resorcin should be applied to the uterus only in the shape of tampons, ointments or, still better, in stick. Applied in this way, Andeer has used it successfully in all uterine troubles that demand it.

II. Diseases of the Intestinal Canal.—The intestines resorb resorcin rapidly and bear it without injury, just as the vagina and urethra, organs rich in epithelium. In cholera infantum, septic enteritis, typhoid and like septic processes, the intestines—and uterus as well—possess a greater tolerance for resorcin than when in a physiological condition.

III. Suppuration and Abscess.—The most concentrated solutions of resorcin can be used in washing out abscesses without manifesting the least reflex reaction. This is also true as to washing out the antrum of Highmore, sinus frontalis, œsophagus, etc., with similar solutions of resorcin. A patient, 50 years old, suffering with an extensive abcess reaching from the last vertebra to the coccyx, and arising from a pleuritic exudation, with several fistulæ, bore admirably daily injections of resorcin. For several days the injections in this case were made several times each day, and in no instance was the least unpleasant reaction observable.

IV. Syphilis.—Privy Counsellor v. Rinecker, in Wuerzburg, has employed resorcin since 1878 in his private practice in syphilitic affections with the best results. He has made the following observations: Resorcin acts promptly and safely in soft and hard chancres, in phagedœna, in fresh localized infiltrations and ulcerations; and in all these cases it should be applied in solid stick or with cosmoline as an ointment.

Andeer recommends a thorough and systematic antisyphilitic treatment with mercury or iodide of potassium in cases of skin syphilides, combined with an energetic application of resorcin locally.

---

*Paralysis and Affections of the Optic Nerve following Injuries of the Head. Leber & Deutschmann, Archives f. Ophthal, XVII.*—I. Leber has very recently called attention to the fact that cases of injury to the head, followed by blindness of one eye or amblyopia presenting the ophthalmological picture of simple atrophy of the optic nerve, are dependent on a lesion of the optic nerve in the region of the foramen opticum. These cases have since that won a new interest through medical jurisprudence. Cases recently communicated by Berlin establish the fact of recent fracture of the walls of the canalis opticus. Leber & Deutschmann communicate ten analogous observations, in which there were violent symptoms after injuries to the head.

Most of the cases were caused by falling from considerable height and striking the head, followed by loss of consciousness and the usual symptoms of the fracture of the base, as hemorrhage from the mouth, nose, ears, and suggillations on the well-known points of predilection.

Blindness of either one or both eyes occurred in all of these instances almost immediately after the accident, and that, too, without there being any demonstrable changes in the eye. In from 2-3 weeks after such injury changes in the fundus begin to show themselves, consisting in a discoloration of the papilla, which gradually grows to be an expressed white atrophy of the optic nerve. The blood-vessels of the retina appear perfectly normal. Only in one case, fall on the forehead, was there total blindness of both eyes. Of the remaining nine cases, in six existed amaurosis, and in three amblyopia in a high degree, with a diminution of the field of vision. In three cases there was a complication with paralysis: twice, with paralysis of the oculomotorius; once, with paralysis of oculomotorius and abducens. In one case there was paralysis of the oculomotorius alone without other symptoms; this disappeared spontaneously in 11 weeks. In one case there was no other symptom than a paralysis of the trochlearis, which disappeared after four weeks.

---

*Two Cases of Rupture of the Bladder. Wochenscrift. No.* 22, 1881. *J. Assmuth.*—In the monograph of M. Bartels there is not a single instance, among 169 cases, of rupture of the bladder caused by pressure of the abdominal muscles from above on the filled bladder. Assmuth's cases are as follows: (1) Man, 32 years old; perceived pain in the region of the bladder while lifting a heavy weight Immediately suppression of urine followed. On endeavoring to empty the bladder only a few drops of blood appeared. Out of unimportant initial symptoms a universal peritonitis developed, from which patient succumbed on the sixth day. The autopsy showed a rupture of the posterior wall of the bladder a little to the right from the middle. (2) Patient, man, 40 years old; came to hospital two days after having lifted a heavy burden, since which time he was unable to urinate. Symptoms of collapse and peritonitis were already present. A catheter was introduced, but only a few drops of blood, mingled with a very small quantity of urine, passed. Death on the sixth day from peritonitis. Autopsy revealed two ruptures of the bladder about, respectively, 2 and 3 centimetres long and situate in the right posterior wall. Assmuth declares that in the future he will not treat similar cases expectatively. The author could find only two analogous cases of intra-peritoneal rupture of the bladder that have recovered, viz.: (1) Case of Walter, Pittsburg, Pa., recovery after introduction of a Sonde a demeure; (2) Case of Morris, Middlesex Hospital, London, recovery following laparotomy.

---

*A New Chinese Remedy for Skin Disease. J. Neumann. Med. Blaetter, Vienna. No.* 27, 1881.—This preparation is a tincture of a root of a plant growing in the interior of Siam. The author not having the entire plant, was unable to classify it. This tincture is used with great success in China and India as a remedy for skin diseases, especially for ringworm. It is applied locally several times a day, and is perfectly harmless. Prof. Vogl has examined the tincture and expressed the opinion that it is made from the root of rhinacanthus communis, an acanthacea growing in Madagascar and East India, which formerly was recommended for herpetic eruptions. Neumann has used it in chronic eczema papulosum and herpes tonsurans vesiculosus. In both of these troubles he cured several cases in two weeks by applying the tincture several times a day. Cases of infiltration of the skin treated with this tincture were not at all benefitted. As to the therapeutic value of the remedy the author expresses himseif reservedly, but considers it worthy of future experiments.

---

*Croupous Inflammation of Steno's Duct. B. Stiller, Wochenschrift, No.* 19, 1881.—Stiller describes a case similar to the one first described by Kussmanl (*Centrbl.*, 1879, *p.* 714), of a man 36 years old, who now and then (3–6 months) was suddenly affected with an enlargement of the parotid, which always came on during mastication. Patient had discovered that by employing digital pressure on the tumor it disappeared rapidly, at the same time a few drops of pus-like substance first exuded from the mouth, followed by an abundant flow of a clear fluid—parotid saliva—out of the mouth. Patient can at any time, when the attack is not present, empty a fluid from the right side (one affected) by simple pressure. Ipscher, in *Wochenschrift*, No. 36, 1879, mentions a case of periodical swelling of the submaxillary gland as a result of croupous inflammation of Wharton's duct, which also appeared during mastication, and could be made to disappear in a like manner by pressure on the swelling.

---

*Treatment of Acne Disseminata and Sycosis. G. Behrend, Wochenschrift, No.* 20, 1881.—B. holds, that even in cases of acne and sycosis when some constitutional anomaly is present, energetic local treatment is necessary, because one efflorescence produces a second. The pressure exercised by an acne nodule is strongest on the surface, thus preventing the secretion of sebaceous matter, and at the same time causing a transudation into the cutaneous glands, which last promotes the production of new nodules. On account of the failure of other means, Behrend has tried and warmly recommends the local measures laid down by Auspitz—in all cases vigorous and continued application of the sharp spoon.

---

*Hydrochinon as an Antiseptic for the Eye. v. Forster, Intelligenz Blatt, No.* 22.—Hydrochinon is peculiarly adapted for the preparation of bandages for the eye. In its antifermentative action it is equal to the phenols, and on account of its solubility in water it far surpasses boracic acid, while it is fully equal to it as regards the irritation following its use. v. Forster obtained excellent results from hydrochinon in infectious troubles of the cornea resting on a parasitic basis. Secretions of the conjunctivas are diminished by it, and in no instance is there the least irritation from it. It's favorable influence on the diphtheritic process is directly attributable to it's antiseptic action.

*Suffocation from Tobacco in Chloroform Narcosis. G. Fischer, Deut. Zeitschrift, XV., p.* 188.—Chloroform was administered to a patient with a compound fracture of the tibia; before the bandage was completed fatal asphyxia took place. At the autopsy a piece of chewing tobacco was found lodged in the vocal chords. It was round, 2.5 ctm. long, 1 ctm. broad, and closed up the glottis entirely Although this is the first case in literature of such results from a chew of tobacco in narcosis, still the author considers it the duty of every physician to examine the mouth of the patient for tobacco before beginning the narcosis.

---

*An Artificial Antiseptic Tympanum. G. Czarda, Med. Presse, Vienna, No.* 20, 1881.—The artificial tympanum is made with a punch, out of silk protective. The protective can be doubled and then strengthened by disks of macintosh, which are to be fixed together by means of collodion and fastened concentric to both sides of the silk protective with a silk string impregnated with boracic acid, or by a fine silver wire. The introduction is made through a guiding tube.

